# Supplementary material for: CleanUpRNAseq: An R/Bioconductor Package for Detecting and Correcting DNA Contamination in RNA-Seq Data
Source: BioTech (Basel). 2024 Aug 3;13(3):30. doi: 10.3390/biotech13030030 (PMC11348166; doi:10.3390/biotech13030030)
Supplement: Supplementary file 1 [file biotech-13-00030-s001.zip › CleanUPRNAseq_SupplementaryMaterials/Figure S1¿CS10.pdf]

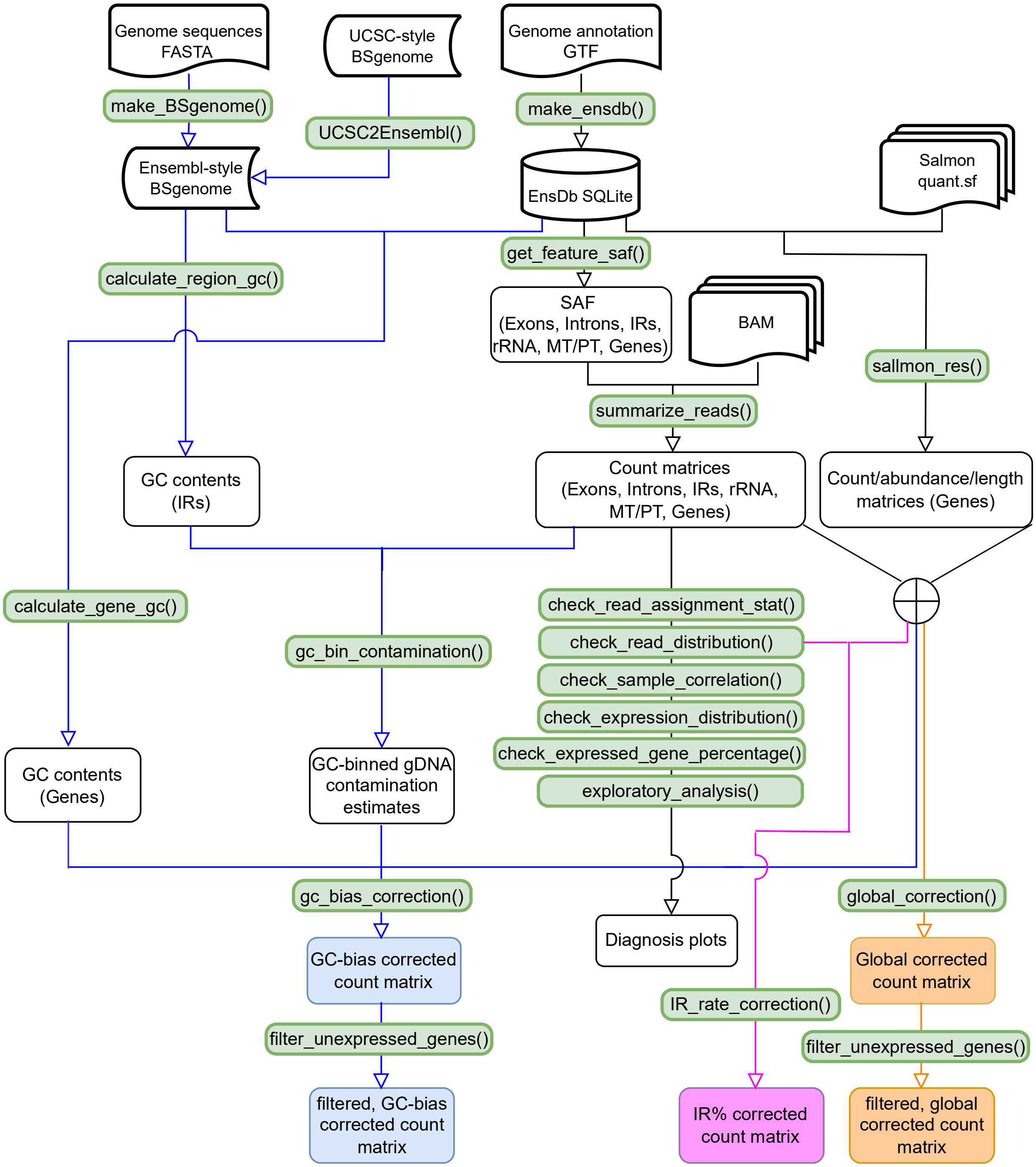

Figure S1. Flowchart of using CleanUpRNAseq to detect and correct for gDNA contamination in unstranded RNA-seq data. The data flow specific to each of the three correction methods—the "IR%" method, the "GC%" method, and the "Global" method—is depicted with arrows in pink, blue, and orange, respectively, while the common data flow among the three methods is illustrated with black arrows. Function calls are highlighted in light green. Gene-by-sample expression matrices corrected by the three methods are highlighted accordingly. For more details, please refer to the Methods and Materials section.

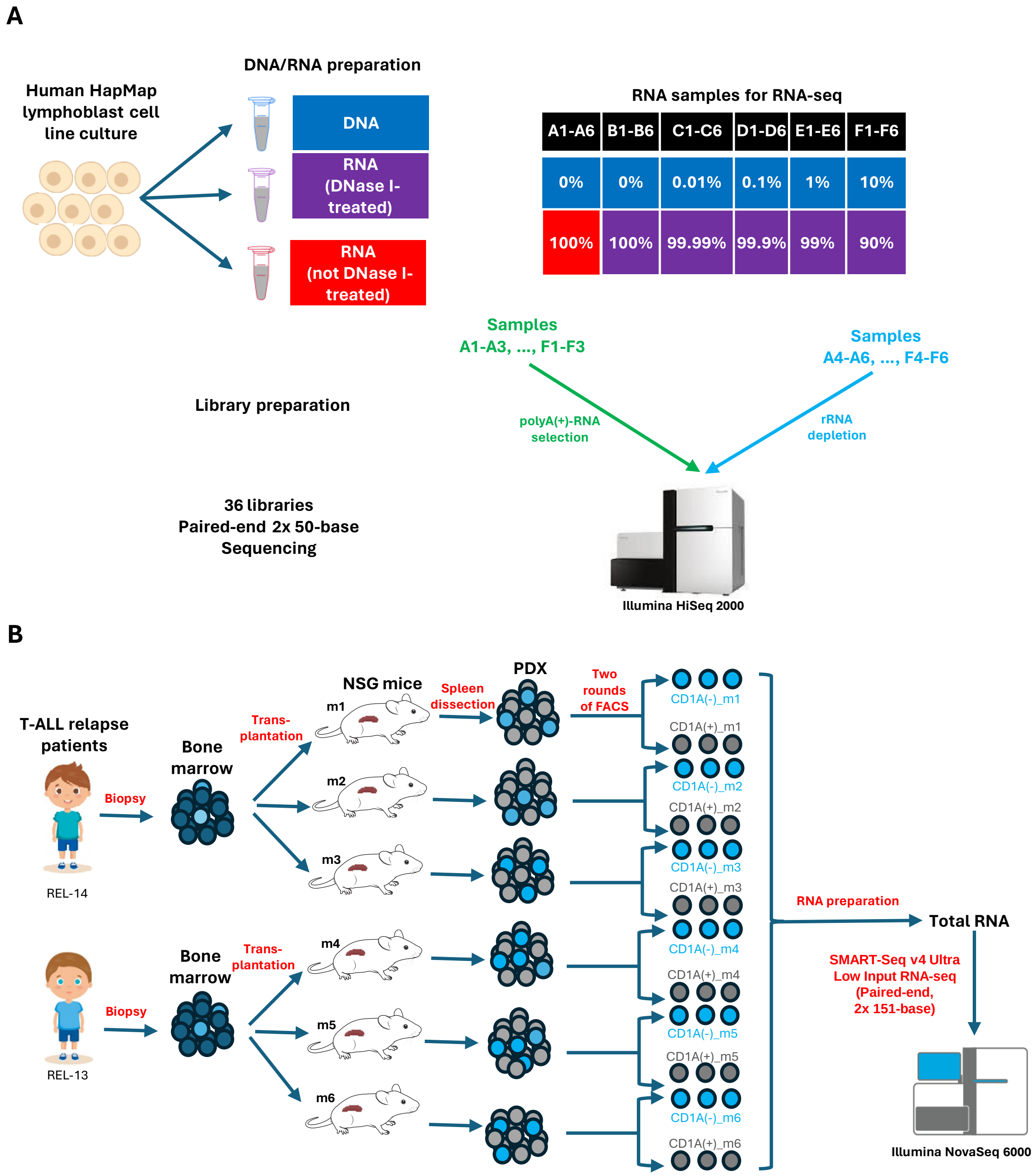

Figure S2. Schematic diagrams illustrating the experimental designs used to generate the two datasets: Datasets I and II. (A) Experimental design used by Li et al to generate RNA-seq data with known levels of gDNA contamination (Dataset I). Total RNA and gDNA were extracted from the culture of the human HapMap lymphoblast cell line. One aliquot of the total RNA was treated with DNase I, while another aliquot was left untreated. Aliquots of DNase I-treated RNA were mixed with varying amounts of gDNA so that the gDNA accounted for 0%, 0.01%, 0.1%, 1%, or 10% of the total nucleic acids (250 ng) by mass (Samples B1-B6, …, F1-F6). These RNA:gDNA mixtures and the RNA sample not treated with DNase I (Sample A1-A6) were used to construct the RNA-seq libraries. RNA-seq libraries were prepared using the polyA(+)-RNA selection method for Samples A1-A3, …, F1-F3, while RNA-seq libraries were constructed using the rRNA depletion method for Samples A4-A6, …, F4-F6, resulting in 36 sequencing libraries. Paired-end reads (2× 50-base) were generated on an Illumina HiSeq2000 platform. (B) Experimental design used by O'Conner et al to generate RNA-seq data with ultra-low RNA input (Dataset II). Briefly, bone marrow biopsies were obtained from two T-ALL relapse pediatric patients and transplanted to six NSG (NOD-scid IL2Rgammanull) mice to generate PDXs. After a period of time, human CD7+CD1A+cells (actively cycling, chemosensitive, T-lineage committed leukemic T cell precursors) and $CD7^{+}CD1A^{-}$ cells (cycle-restricted,chemoresistant, multi-lineage leukemia-initiating T cell precursors) were sorted out from each of the PDXs and used for RNA-seq assays with the SMART-Seq® v4 Ultra® Low Input RNA Kit for Sequencing (Takara Bio, Japan). Paired-end reads (2× 151-base)were generated on an Illumina NovaSeq 6000 platform.

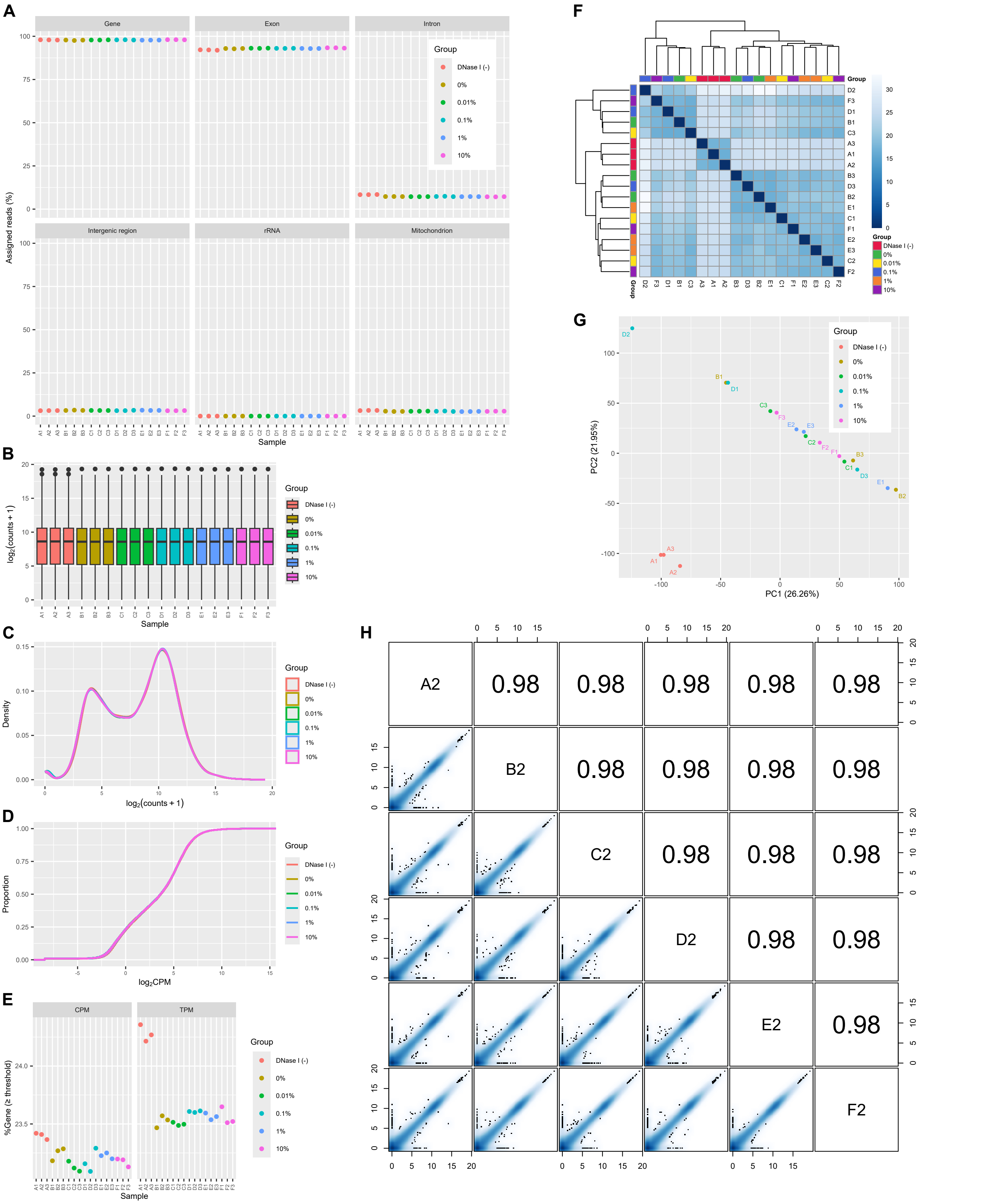

Figure S3. Diagnostic plots reveal little gDNA contamination in RNA-seq data generated with the polyA(+)-RNA selection method. (A) Dot plots showing percentages of reads mapping to different genomic features: genes, exons, introns, intergenic regions, rRNA exons, and the mitochondrial genome. (B) Box plots showing sample-level gene expression distributions. The gene-by-sample count matrix was normalized using the DESeq2's median of ratios method, followed by log transformation with a pseudocount of 1 added to each value. (C) Density plots showing sample-level gene expression distributions. The count matrix was normalized and transformed as in (B). (D) Empirical cumulative distributions of sample-level gene expression. The gene-by-sample count matrix was converted to a gene-by-sample CPM matrix using the cpm function of the edgeR package. A pseudocount equal to one-tenth of the minimal CPM value in the matrix was added to the CPM matrix, followed by log transformation. The resulting matrix was used to generate the empirical cumulative distributions of sample-level gene expression using the stat_ecdf function of the ggplot2 package. The empirical cumulative distributions of sample-level gene expression reveal the proportions of genes (y-axis) with $\log_2$CPM equal to or less than any given value (x-axis). (E) Dot plots showing percentages of genes with expression levels above one CPM (left) and one TPM (right). (F) Smooth scatter plots and Pearson correlation coefficients showing gene expression similarities between sample pairs. The count matrix was normalized and transformed as in (B). (G) Hierarchically clustered heatmap showing Euclidean distances between pairs of gene expression profiles. The gene-by-sample count matrix was normalized as in (B) and transformed using the vst function in the DESeq2 package. The heatmap was generated using the pheatmap function of the pheatmap package. The color key shows the scale of distances. (H) PCA score plots showing variability in gene expression profiles. The transformed matrix in (G) was scaled, centered, and used for PCA with the prcomp function in the base library of R.

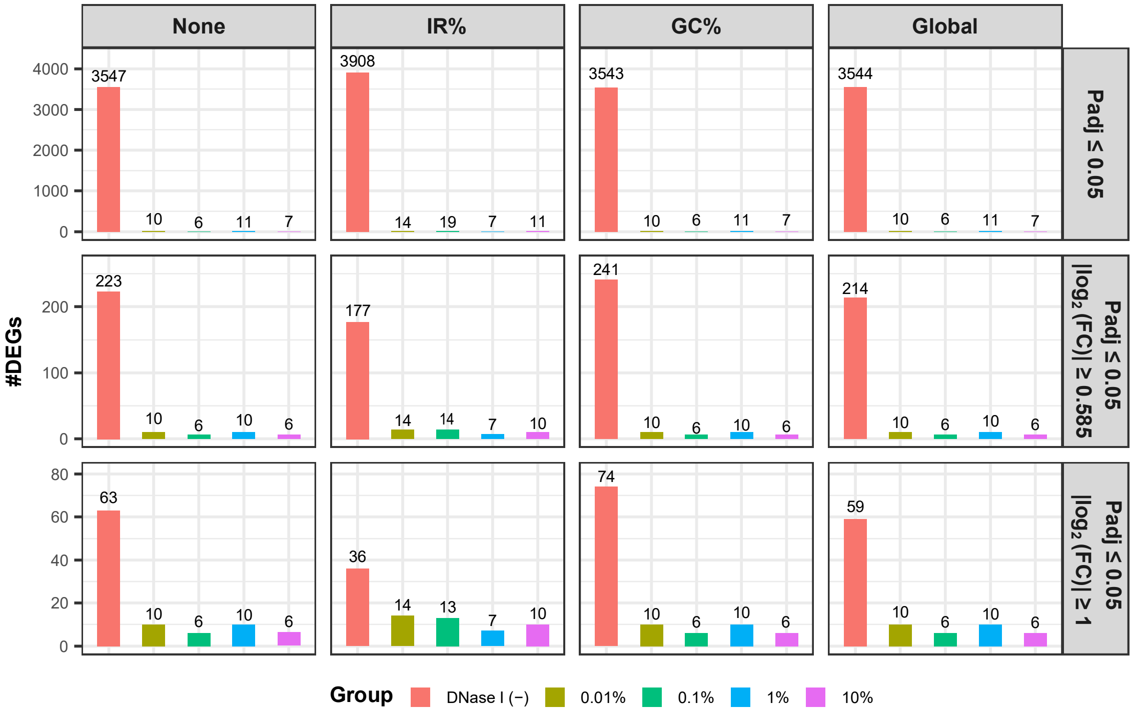

Figure S4. Correction for gDNA contamination barely reduces false positive DEGs in RNA-seq data generated by the polyA(+)-RNA selection method. Please refer to the legend of Figure 1 for an explanation of labels. The group of DNase I-treated RNA samples with no added gDNA (Samples B1-B3) was used as the control group. Differential expression analyses were performed between this control group and RNA samples untreated with DNase I (DNase I (−)), or DNase I-treated RNA samples with varying levels of added gDNA (0.01%, 0.1%, 1%, 10%). "None" and "IR%" signify the use of raw count matrix as the argument of the countData parameter of the DESeqDataSetFromMatrix function, while "GC%" and "Global" denote the utilization of "GC%" and "Global" method-corrected count matrices, respectively. Regarding the design matrix specification, "IR%" utilizes "design = ~ group + IR%", while the other three employ "design = ~ group". To assess the correction effects, three progressively stringent criteria were used for identifying DEGs: adjusted *p*-value ≤ 0.05 (**up**); adjusted *p*-value ≤ 0.05 and $|\log_2\text{FoldChange}| \geq 0.585$ (**middle**); adjusted *p*-value ≤ 0.05 and $|\log_2\text{FoldChange}| \geq 1$ (**bottom**).

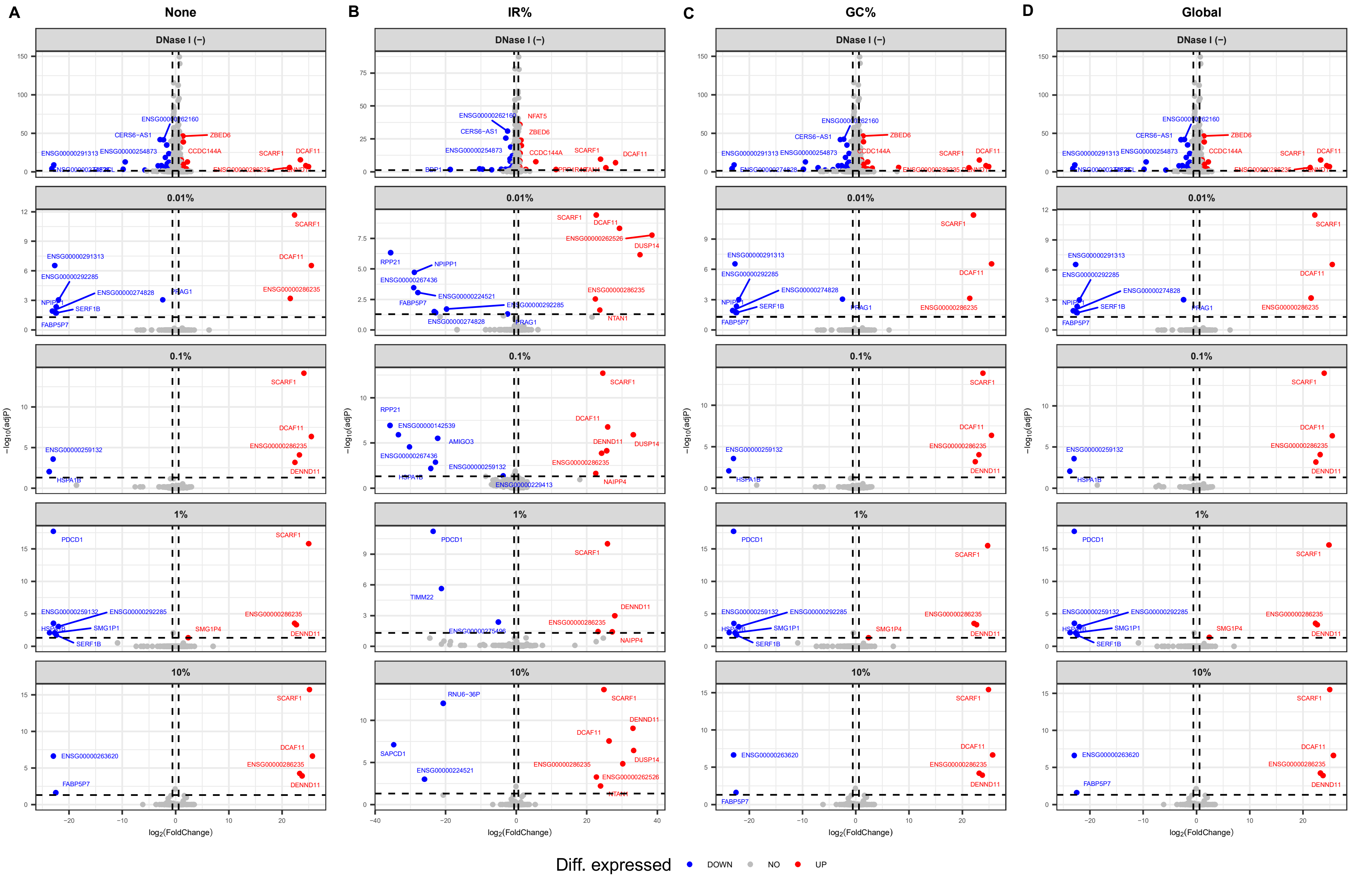

Figure S5. Volcano plots showing DEGs in RNA-seq data generated by the polyA(+)-RNA selection method before and after gDNA contamination correction. Please refer to the legend of Figure 1 for an explanation of the labels. The group of DNase I-treated RNA samples with no added gDNA (Samples B1-B3) was used as the control for differential expression analysis. Differential expression analyses were performed between this control group and RNA samples untreated with DNase I (DNase I (−)), or DNase I-treated RNA samples with varying levels of added gDNA (0.01%, 0.1%, 1%, or 10%). “None” and “IR%” signify the use of raw count matrix as the argument of the countData parameter of the DESeqDataSetFromMatrix function, while “GC%” and “Global” denote the utilization of “GC%” and “Global” method-corrected count matrices, respectively. Regarding the design matrix specification, “IR%” utilizes “design = ~ group + IR%”, while the other three employ “design = ~ group”. Gene were categorized into three groups, down-regulated DEGs (DOWN, blue), non-DEGs (NO, gray), and up-regulated DEGs (UP, red), with a cutoff of adjusted *p*-value ≤ 0.05 and $|\log_2 \text{FoldChange}| \geq 1$. (A) Volcano plots showing DEGs between each gDNA-containing group and the control group without any correction of gDNA contamination. (B-D) Volcano plots showing DEGs between each gDNA-containing group and the control group without any correction of gDNA contamination after correction of gDNA contamination with the “IR%” (B), “GC%” (C), and “Global” (D) methods, respectively.

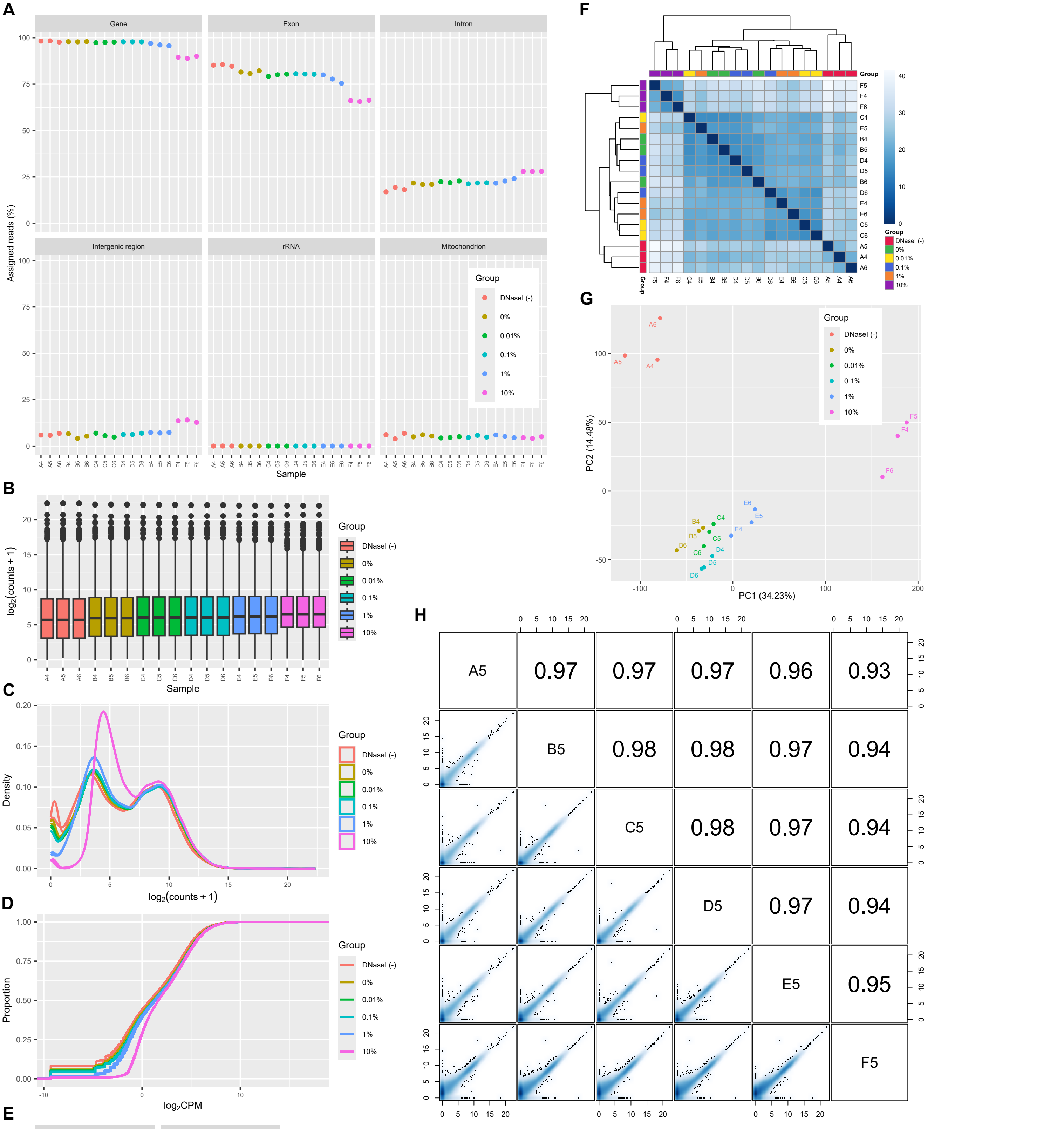

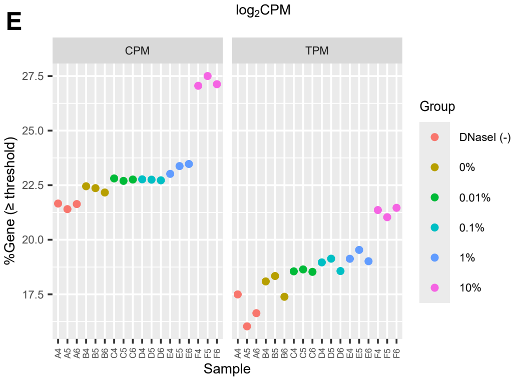

Figure S6. Diagnostic plots reveal gDNA contamination in RNA-seq data generated with the rRNA depletion method. (A) Dot plots showing percentages of reads mapping to different genomic features: genes, exons, introns, intergenic regions, rRNA exons, and the mitochondrial genome. (B) Box plots showing sample-level gene expression distributions. The gene-by-sample count matrix was normalized using the DESeq2's median of ratios method, followed by log-transformation with a pseudocount of 1 added to each value. (C) Density plots showing sample-level gene expression distributions. The count matrix was normalized and transformed as in (B). (D) Empirical cumulative distributions of sample-level gene expression. The gene-by-sample count matrix was converted to a gene-by-sample CPM matrix using the cpm function of the edgeR package. A pseudocount equal to one-tenth of the minimal CPM value in the matrix was added to each entry in the CPM matrix, followed by log transformation. The resulting matrix was used to generate the empirical cumulative distributions of sample-level gene expression using the stat_ecdf function of the ggplot2 package. The empirical cumulative distributions of sample-level gene expression rea the proportions of genes (y-axis) with $\log_2$CPM equal to or less than any given value (x-axis). (E) Dot plots showing percentages of genes with expression levels above one CPM (**left**) and one TPM (**right**). (F) Smooth scatter plots and Pearson correlation coefficients showing gene expression similarities between sample pairs. (G) Hierarchically clustered heatmap showing Euclidean distances between pairs of gene expression profiles. The gene-by-sample count matrix was normalized as in (B) and transformed using the vst function in the DESeq2 package. The heatmap was generated using the pheatmap function of the pheatmap package. The color key shows the scale of distances. (H) PCA score plots showing variability in gene expression profiles. The transformed matrix in (G) was scaled,centered, and used for PCA with the prcomp function in the base library of R.

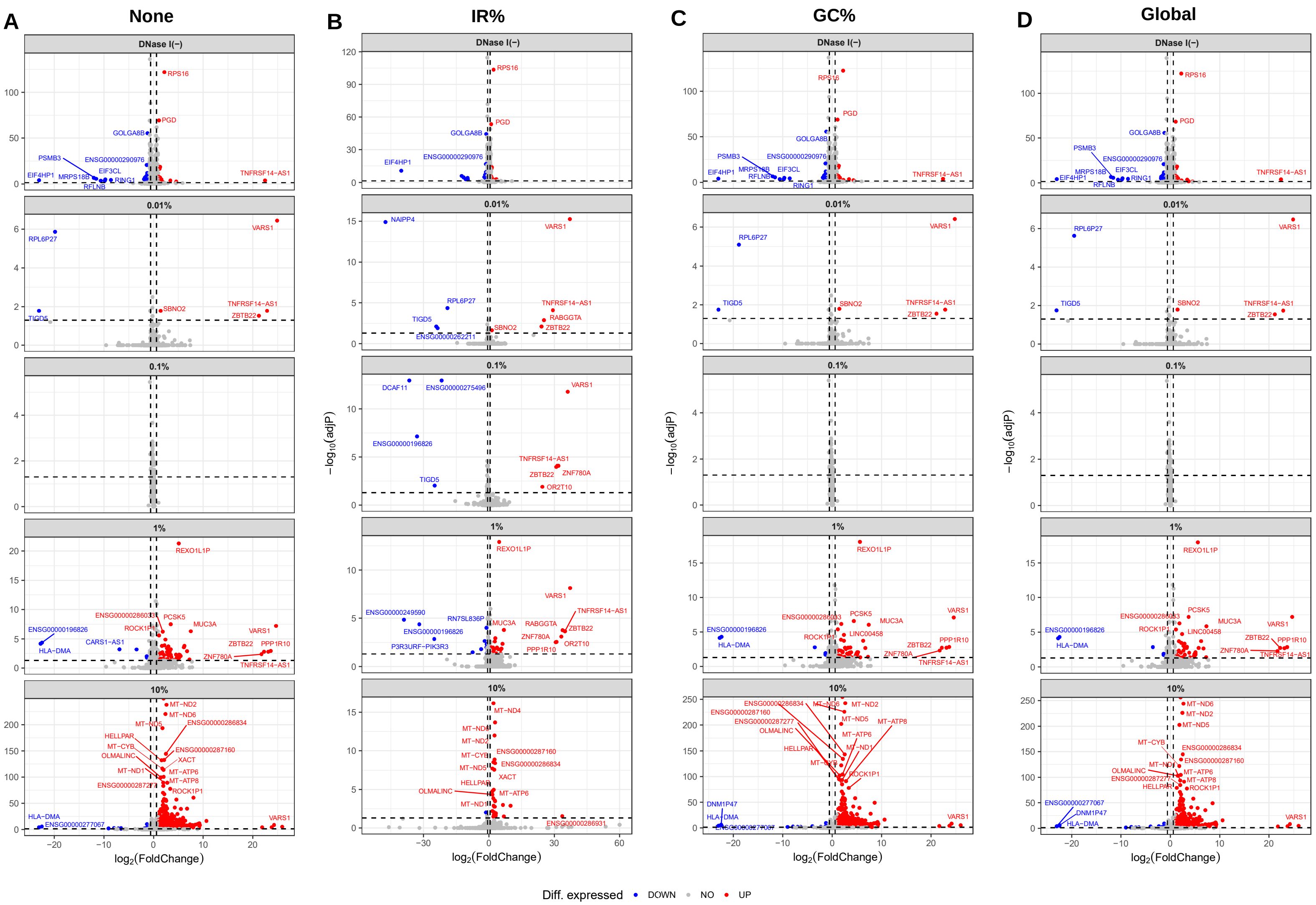

Figure S7. Volcano plots showing DEGs in RNA-seq data generated from rRNA-depletion method before and after gDNA contamination correction. Please refer to the legend of Figure 1 for an explanation of the labels. The group of DNase I-treated RNA samples with no added gDNA (Samples B4-B6) was used as the control group. Differential expression analyses were performed between this control group and RNA samples untreated with DNase I (DNase I (-)), or DNase I-treated RNA samples with varying levels of added gDNA (0.01%, 0.1%, 1%, or 10%). “None” and “IR%” signify the use of raw count matrix as the argument of the countData parameter of the DESeqDataSetFromMatrix function, while “GC%” and “Global” denote the utilization of “GC%” and “Global” method-corrected count matrices, respectively. Regarding the design matrix specification, “IR%” utilizes “design = ~ group + IR%”, while the other three employ “design = ~ group”. Gene were categorized into three groups, down-regulated DEGs (DOWN, blue), non-DEGs (NO, gray), and up-regulated DEGs (UP, red), with a cutoff of adjusted *p*-value ≤ 0.05 and | $\log_2$FoldChange| ≥ 1. (A) Volcano plots showing DEGs between each gDNA-containing group and the control group without any correction of gDNA contamination. (B-D) Volcano plots showing DEGs between each gDNA-containing group and the control group without any correction of gDNA contamination after correction of gDNA contamination with the “IR%” (B), “GC%” (C), and “Global” (D) methods, respectively.

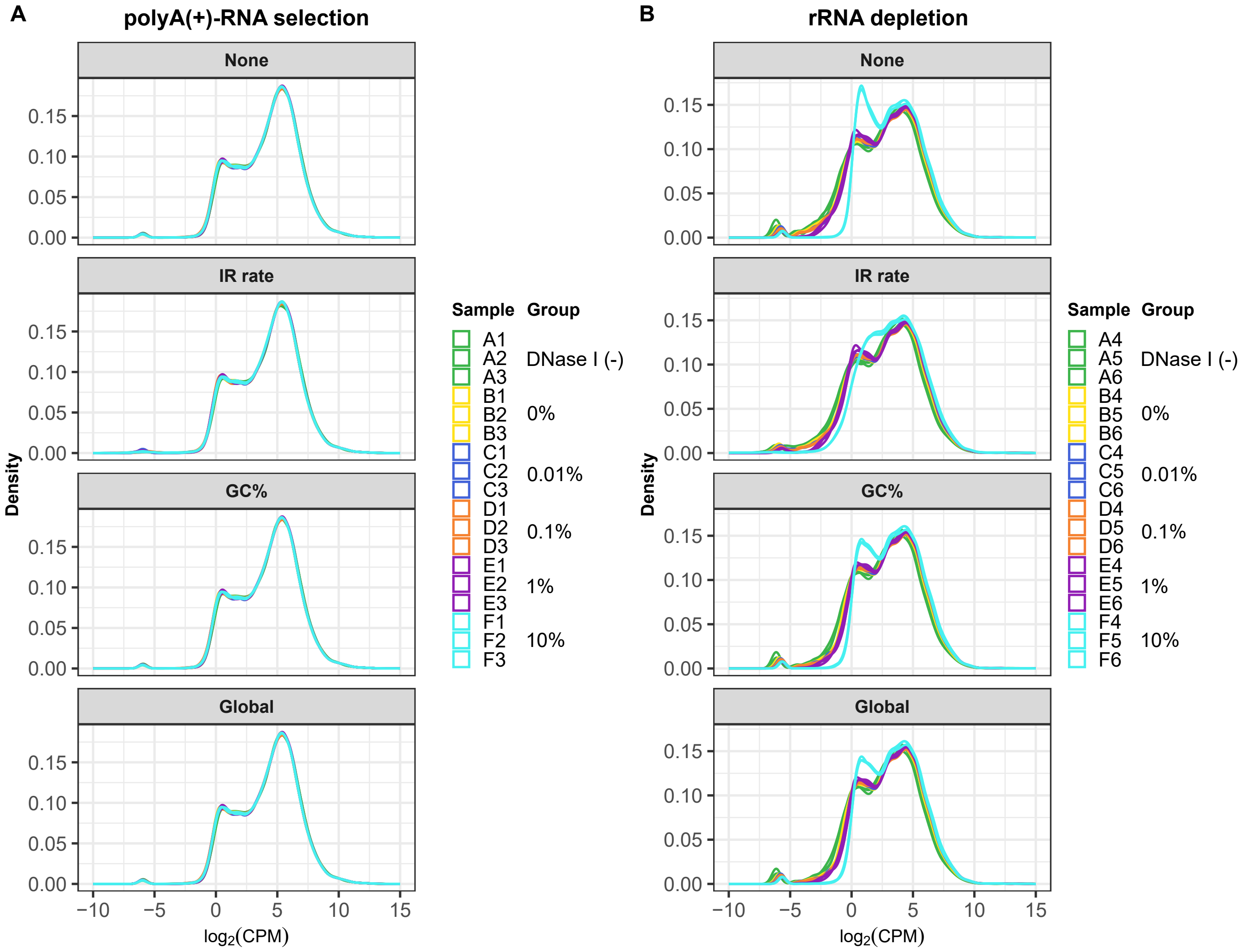

Figure S8. Density plots depicting gene expression distributions before and after gDNA contamination correction in RNA-seq Dataset I. Please refer to the legend of Figure 1 for an explanation to the labels. The corresponding count matrix, whether raw ("None", and "IR%") or corrected ("GC%", and "Global"), was transformed into a $\log_2$CPM matrix using the voom function of the limma package. The corresponding $\log_2$CPM matrices were used for density plots, except in the case of the "IR%" method, which is outlined below. The $\log_2$CPM matrix ("IR%") was used to fit linear models for differential expression analysis using the lmFit function of the limma package with the model matrix specified as "~group + IR%". The estimated parameters for the covariate were used to correct $\log_2$CPM matrices through matrix operations. subsequently employed for density plots. (A) Density plots depicting gene expression distributions before and after gDNA contamination correction in RNA-seq data generated with the polyA(+)-RNA selection method. (B) Density plots depicting gene expression distributions before and after gDNA contamination correction in RNA-seq data generated with the rRNA depletion method.

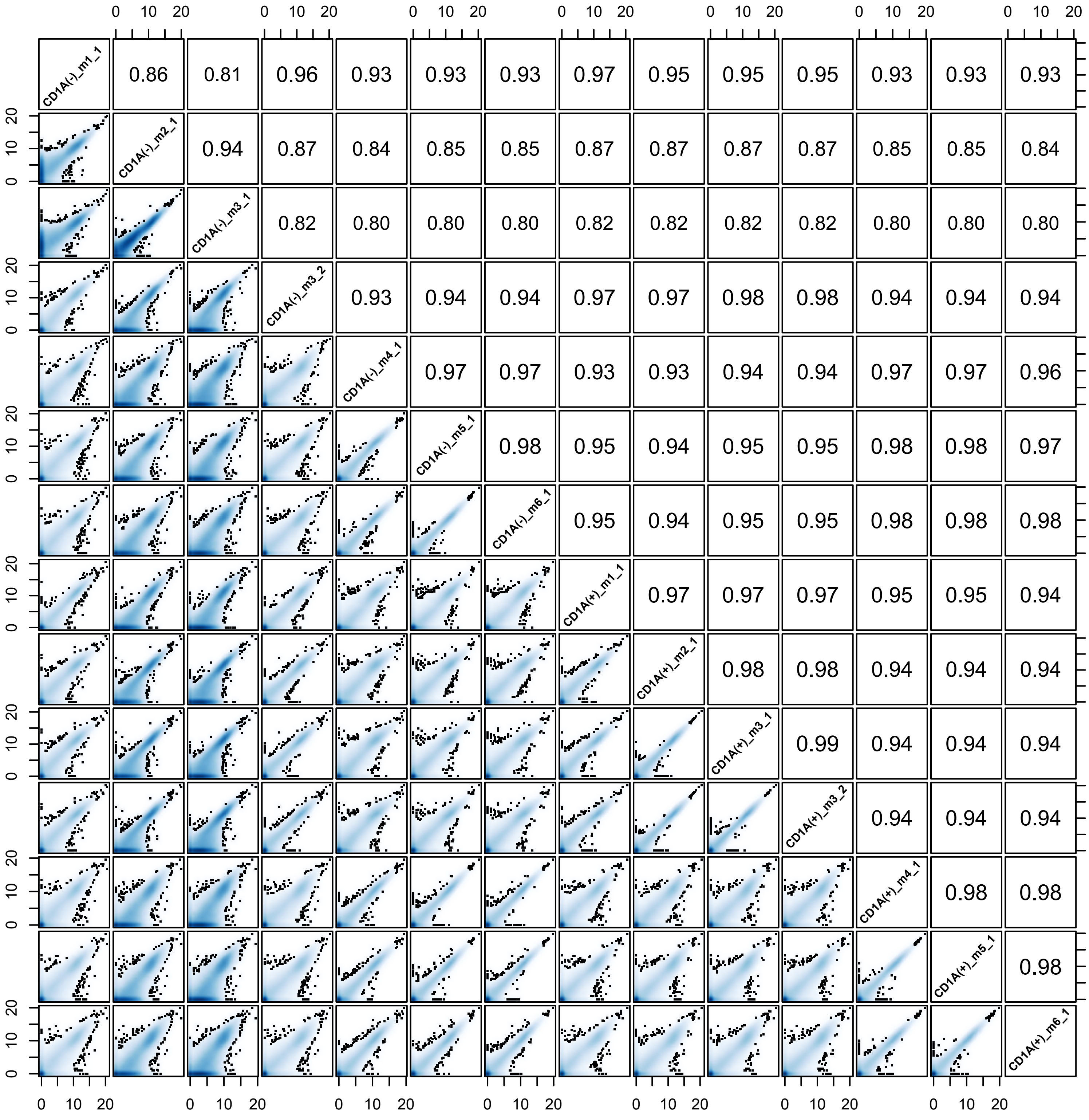

CD1A(-)_m1_1
CD1A(-)_m2_1
CD1A(-)_m3_1
CD1A(-)_m3_2
CD1A(-)_m4_1
CD1A(-)_m5_1
CD1A(-)_m6_1
CD1A(+)_m1_1
CD1A(+)_m2_1
CD1A(+)_m3_1
CD1A(+)_m3_2
CD1A(+)_m4_1
CD1A(+)_m5_1
CD1A(+)_m6_1
0.86 0.81 0.96 0.93 0.93 0.93 0.97 0.95 0.95 0.95 0.93 0.93 0.93
0.94 0.87 0.84 0.85 0.85 0.87 0.87 0.87 0.87 0.85 0.85 0.84
0.82 0.80 0.80 0.80 0.82 0.82 0.82 0.82 0.80 0.80 0.80
0.93 0.94 0.94 0.97 0.97 0.98 0.98 0.94 0.94 0.94
0.97 0.97 0.93 0.93 0.94 0.94 0.97 0.97 0.96
0.98 0.95 0.94 0.95 0.95 0.98 0.98 0.97
0.95 0.94 0.95 0.95 0.98 0.98 0.98
0.97 0.97 0.97 0.95 0.95 0.94
0.98 0.98 0.94 0.94 0.94
0.99 0.94 0.94 0.94
0.94 0.94 0.94
0.98 0.98
0.98
0 10 20

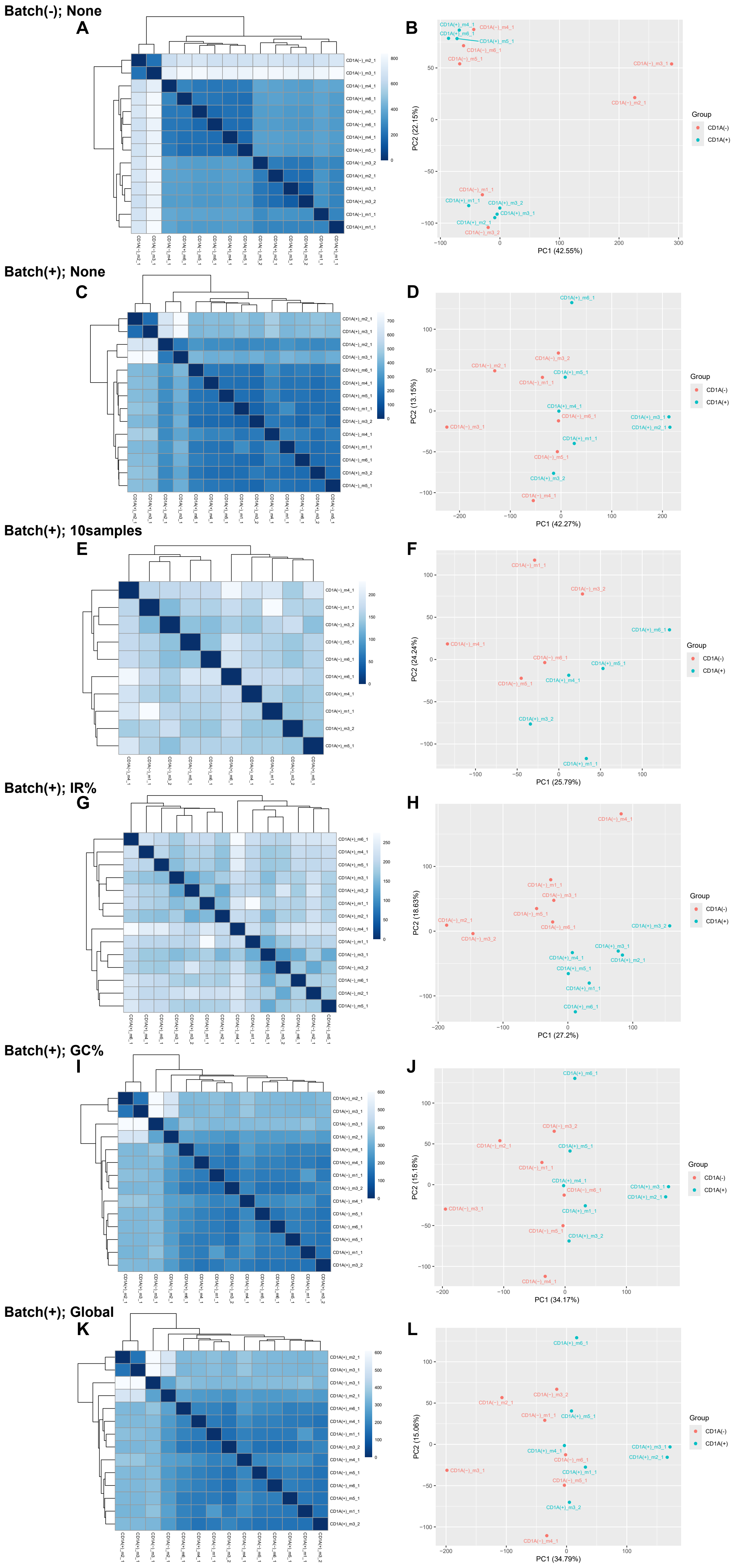

Figure S10. Diagnostic plots for assessing the impact of correcting for gDNA contamination and batch effects in Dataset II. Please refer to the legends of Figures 3 and 4 for data usage and an explanation of the labels. The corresponding count matrix, whether raw ("Batch(−);None", "Batch(+);None", "Batch(+);10samples", and "Batch(+);IR%") or corrected ("Batch(+);GC%", and "Batch(+);Global"), was transformed into a log_2CPM matrix using the voom function of the limma package. These log_2CPM matrices were used to fit linear models for differential expression analysis using the lmFit function of the limma package with the model matrices specified as "~group" ("Batch(−);None"), "~group + batch" ("Batch (+);None"), "~group +batch + IR%" ("Batch(+);IR%"), "~group + batch" ("Batch(+);GC%"), "~group + batch" ("Batch(+);Global"). The estimated parameters for covariates,where appropriate, were used to correct log_2CPM matrices through matrix operations, for visualization. The raw (A) and corrected log_2CPM matrices (C, E, G, I, and K) were used for hierarchical clustering. (A, C, E, G, I, and K). Hierarchically clustered heatmap showing Euclidean distances between pairs of gene expression profiles before and after correction for gDNA contamination as well as batch effects. The heatmaps were generated using the pheatmap function of the pheatmap package. The color key shows the scale of distances. (B, D, F, H, J, and L) PCA score plots showing variability among gene expression profiles. The transformed matrices used in (A, C, E, G, I, and K) were scaled, centered, and then used for PCA with the prcomp function in the base library of R.
